# Morphometric Measurements and Muscle Atrophy Scoring as a Tool to Predict Body Weight and Condition of Horses

**DOI:** 10.3390/vetsci10080515

**Published:** 2023-08-09

**Authors:** Nadine Urbanek, Qendrim Zebeli

**Affiliations:** Institute of Animal Nutrition and Functional Plant Compounds, Department for Farm Animals and Veterinary Public Health, University of Veterinary Medicine Vienna, Veterinaerplatz 1, 1210 Vienna, Austria; nadine.urbanek@gmx.at

**Keywords:** body weight, body condition score, crest neck score, muscle atrophy score, over-condition, morphometric measurements

## Abstract

**Simple Summary:**

Estimations of the body condition and body weight are important in horses to prevent disease and also to maintain the performance, fertility, and physical and mental stress tolerance of the animal. Yet, the estimation of body condition and body weight under practical circumstances is not easy. This research developed models that help in the prediction and estimation of body weight and body condition score of horses under practical conditions using common morphometric measurements of the body and evaluations of the Cresty Neck Score and Muscle Atrophy Scoring System; hence, these take into account the size of the animal, its abdominal extension and filling, regional fat adiposity, age, and degree of the muscularity.

**Abstract:**

Accurate estimation of body weight (BW) and condition (BCS) is important in the equine practice. The main goal of this research was to develop models for the prediction of BW and BCS of horses in the practice using both common morphometric measurements and measurements of Cresty Neck Score (CNS) and Muscle Atrophy Scoring System (MASS) as a measure of muscularity. Our model showed that the BW of horses could be predicted with high reproducibility (concordance correlation coefficient = 0.97), accuracy (0.99), and precision (0.97) using the morphometric measurements of the height at withers, circumference of the chest, cane circumference, body length, and body circumference as well as the BCS, CNS, and muscle atrophy score of the hindlimbs. The stepwise multiple regression analysis revealed that the BCS of horses can be predicted with the data of parameters such as age, body length and an index consisting of measurements of the body circumference to height of withers, and the atrophy of the neck. Future research should use larger cohorts of animals to validate the findings of this study.

## 1. Introduction

Obesity is a growing problem in horses worldwide [1,2,3]. On the other hand, studies indicate that very often over-conditioned horses are not recognized as such by their owners [4]. An accurate estimation of body condition and body weight (BW) is important to prevent diseases [5,6] and also to maintain performance [7], fertility [1], and physical and mental stress tolerance [2] of the horse. 

The most common way used to determine the body condition of a horse is the body condition score (BCS) developed by Henneke [8], which allows a classification of the subcutaneous body fat in various regions of the body. This system was developed to determine the body condition of pregnant Quarter Horse mares by palpating different regions such as the neck, the wither, the shoulder, the rip, the hip, and the tailhead, which—depending on fat depots—are categorized from 1 (extremely thin) to 9 (extremely fat). Over time, more systems have been developed like a reduction to six scores (from 0 to 5) by Carrol [9] or the adaptation to Warmblood horses by Schramme [10]. The latter characterizes the fat status of the crest and takes into account more details of the hip region. More recent research has also suggested the Cresty Neck Score (CNS) to differentiate between the general and regional adiposity [11]. Accordingly, the crest is categorized from 0 (no visual crest) to 5 (very big crest, falling to one site) [12]. 

Nevertheless, all the systems ignore the degree of muscularity of the animal. In horses, muscularity is an indicator of health [13], aging [14], as well as training and nutritional status [15,16]. To classify the muscularity in horses, the Muscle Atrophy Scoring System (MASS) has been suggested, whereby the muscles of the neck, the back, and the hindlimbs are considered from both sides and graded from 1 (no atrophy) to 4 (severe atrophy) [17]. Incorporating the MASS in the BSC system would further enhance the evaluation of the body condition and of the BW of the horses. Indeed, like BCS, the estimation of BW of the horse is important for dose medications and also to be able to calculate the exact diet based on the specific needs of the individual in terms of energy and nutrients [18]. For horses, there is no standardized normal BW; so, to establish the body condition and nutritional status, multiple weighing procedures must occur to observe the BW change [10]. In contrast to the BCS, where fat depots are determined, the BW also includes many more factors. For example, the musculature accounts for around 40% of the BW [9,18], which is more than the gastrointestinal tract filling (up to 20%) [9], the bones (approximately 15% of BW), the inner organs and the skin (4%), as well as the blood (8%) [19]. Muscle volume is knowingly heavier than the same volume of body fat. A survey study with owners showed that the BW of horses was around 52 kg off the actual BW for Warmblood and Coldblood breeds [20].

In practice, evaluation of the BW of the horse can either be performed with a scale or estimated by various morphometric measurements. Indeed, since many stables do not have a scale available, the estimation of BW is commonly used. For this purpose, several models have been suggested that use morphometric measurements of the body of the horse—most often the circumference of the neck, chest, and cane as well as the body length and height at the withers [9,20,21,22,23,24].

The main goal of this research was to develop models for the prediction of BW and BCS of horses in practice using both the common morphometric measurements and the CNS, BCS, as well as MASS as a measure of muscularity. The accuracy of the morphometric methods to estimate the BSC and BW of horses was tested by comparing them with measurements obtained with an animal scale as a gold standard. We also evaluated how well horse owners estimate the body condition as well as the MASS of their animal after the owners were asked through a questionary about their estimation of the body condition as well as their feeding practices. 

## 2. Materials and Methods

### 2.1. Measurements of Body Weight, Body Condition Score, Cresty Neck Score, and Muscle Atrophy Scoring in Horses

Forty privately owned horses from the province of Lower Austria were recruited for this study. The animals were weighted with an animal scale (Model horse scale PW 1500 from BOSCHE, L × W × H: 201 × 112 × 11 cm). To do so, the horses were guided on by the owners and the result was read after loading all limbs for five seconds and standing still; ten morphometric measurements were taken. It was also ensured that, after every horse, the scale was calibrated to zero before the next horse was guided on. The inclusion criteria for the horses in this study were a height at the withers of at least 1.4 m, in order to exclude ponies and miniature horses and, thus, reduce the size of the range; an age above six years was chosen, as young horses have not yet completed their growth phase and their body condition can still change considerably. Furthermore, all types of housing were taken into account, including stalls and open and loose stalls, and they were allowed to belong to any performance category (leisure, sport, and boarding). 

Additional to BCS measurements according to Henneke [8], where the regions on both sides were scored and palpated and categorized from 1 (extremely thin) to 9 (extremely fat), the CNS of both sides of the neck were also evaluated according to Carter [12] by manual palpation and scored form 0 (no visible crest) to 5 (big crest falling to one side). The muscle atrophy of the neck and rear legs was also scored, according to Herbst [17], by firstly answering questions to classify if the adipose or lean table comes to use. Afterwards, the neck and hind legs were assessed separately, on both sides, by palpation and visualization and scored from 1 (no atrophy) to 4 (severe atrophy).

Among forty horses, there were 15 mares and 25 geldings, with an age range from 7 to 27 years (mean 14.5 years, median 13.5 years). When the measured horses were divided into two age groups, there were 26 horses under the age of 18 years (i.e., considered as young horses) and 14 of the animals had an age above the inclusive 18 years (considered as representative of rather older horses). Regarding the breeds, there were 10 Warmbloods, 5 Coldbloods, 4 Cobs, 4 Quarter Horses, 2 Thoroughbreds, 2 PRE, 2 Lipizzaner, and 11 Mix-breed horses. Regarding the form of housing, 67.5% of the horses lived in an open stable, 40% were kept in a stall, and only 2.5% had a paddock stall. 

### 2.2. Morphometric Measurements

**Figure 1:**
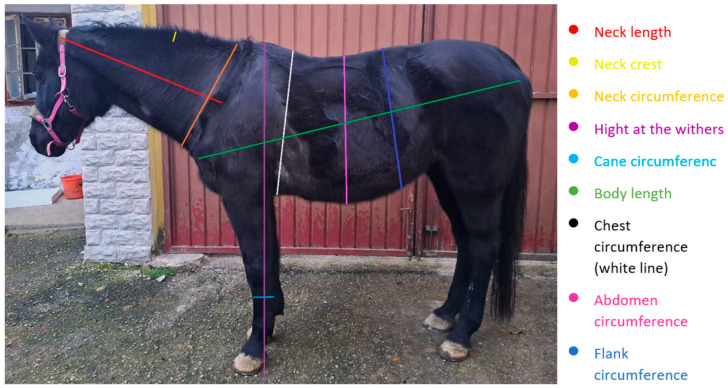
Depiction of morphometric measurements (except the body circumference) shown on the horse.

The morphometric measurements performed are shown in Figure 1. All measurements, except the body length and body circumference, were taken with a 3 m plastic measuring tape with a width of 15 mm. The other two measurements were taken using a 20 m plastic measurement tape with a width of 14 mm. 

The height of the withers was taken with an aluminum stock measure with integrated spirit level, which allowed a height measurement between 1 and 1.80 m. In all measurements, care was taken to ensure that the horse loaded all four limbs equally, and the execution followed the instructions by Schramme [10]. 

The neck length was detected in the neutral position of the neck on the left side, in a straight line from the *Ala Atlantis* to the middle front edge of the scapula (Figure 1). To measure the neck circumference, the tape was placed at the end of the neck, in front of the chest and around the entire neck, and read under slight tension. The crest of the neck was first palpated in neutral position, then measured in the middle of the neck from the hairline of the mane ridge to the dorsal line of the musculature. The height of the withers was taken using an aluminum stick, which was placed straight on the ground, and the highest point was measured using the right-angled siding arm. For chest circumference, the tape measure was placed behind the withers and a hand’s width from the belt position around the horse and read at maximum expiration. The abdominal circumference was measured at the widest point of the trunk and read at maximum expiration. To detect the flank circumference, the tape was applied caudally to the last rib, around the horse, and read at the maximal expiration. The cane circumference was measured on the left weight-bearing limb in the upper third of the cane. For body length, the measuring tape was placed and read from the bone point of the *Pars cranialis* of the *Tuberculum majus* of the humerus to the posterior end of the bone of the *Tuber ischiadicum*. This was aligned with the anterior bone point, applied, and then held by an assistant until the measurement result was read. For the body circumference, the tape measure was again anchored to the *Pars cranialis* of the *Tuberculum majus humeri* and held with the aid of a second person. The tape measure was then passed to the posterior end of the *Tuber ischiadicum* and placed below the tail on the other side; from there, it was passed over the *Tuber ischiadicum* on the other side, forward over the *Pars cranialis* of the *Trochantor majus* across the chest, and back to the starting point, where the result was noted.

### 2.3. Estimated Parameters and Comparison with Models from the Literature

Based on the morphometric measures taken, we estimated both the BW and BCS of the horses using the most recent models from the literature including the following:

1. Body weight (kg) = −1160 + 1.538 × Body circumference + 1.487 × Neck circumference + 2.594 × Height at withers + 13.631 × Body Condition Score + 1.336 × Chest circumference + 6.226 × Cane circumference [24];
2. Body weight (kg) = [Chest circumference^1.528^ ×Body length^0.574^ × Height at withers^0.246^ × Neck circumference^0.261^]/1.209 (Warmblood) [20];
3. Body Condition Score = [8.1493 × (((Chest circumference + Neck circumference)/2)/Height at the withers (cm/cm))] − 2.7572 [25].

### 2.4. Questionnaire Study

In order to obtain deeper information about the horses, their feeding, and to evaluate the extent to which the owners were able to estimate the BW and body condition of their horses, the horse owners were first asked to fill a relevant questionnaire. 

### 2.5. Statistical Analysis

For the development of the models of the BW and BCS based on the morphometric measurements, the Statistical Analysis Software (SAS, Version 9.4., SAS Institute Inc., Cary, NC, USA) was used. We firstly employed stepwise multiple regression analysis with the Regression Procedure (PROC REG) of SAS, using the actual BW or the actual BCS of the horses as the dependent variable. As the independent variables, different morphometric measurements were tested. By removing the non-significant variables (*p* > 0.10) and those with a variance-inflation factor (VIF) of higher than 10, it was ensured that only significant variables without their own correlation or with several strongly correlating predictors (multicollinearity) were used. To check the quality of the models, various parameters were used, including the coefficient of determination (R^2^); the root mean square error (RMSE); the significance level; and for the estimation of body measures, the concordance correlation coefficient (CCC) according to Lin [26]. For the comparison of the models, we used the CCC analysis in PROC IML of SAS, which provides an integrated measure of precision and accuracy (CCC = *r* × *C_b_*, whereby *r* stands for Pearson’s correlation coefficient as a measure of precision, and *C_b_* is the bias correction factor, standing for accuracy). *C_b_* indicates how far the regression line deviates from the ideal line y = x, so that *C_b_* ranges from 0 to 1, whereby 1 indicates full agreement with the ideal line. Other measures to evaluate the accuracy were the scale shift (μ) and location shift (*v*), whereby μ expresses the difference in standard deviation between the measured and estimated values, and *v* indicates under- or overpredictions, where a negative *v* value indicates an overprediction and a positive value indicates underprediction [27]. The measures of accuracy and estimation, including the CCC and its 95% confidence intervals, were computed. According to Hinkle et al. [28], the CCC were interpreted as follows: 0.00–0.30 as negligible; 0.30–0.50 as low; 0.50–0.70 as moderate; 0.70–0.90 as high; and 0.90–1.00 as substantial.

## 3. Results

### 3.1. Data of Body Weight, Morphometric Measurements, and Body Condition Score

The data of descriptive statistics of the BW and morphometric measurements including the span, mean and median, as well as standard deviation are shown in Supplementary Table S1. The BCS showed that 55% of all horses (*n* = 22) had a score > 5, 37% (*n* = 15) had an optimal score of 5, and only 7.5% (*n* = 3) of horses were too thin, with a score of 4 out of 9. Looking at the CNS of all horses, most of them (62.5%; *n* = 25) show a CNS of 2 out of 5 (2/5), meaning a noticeable appearance of the crest, which can easily be cupped in one hand and bent from side to side. The other horses (17.5%, *n* = 7) had a score of 3 out of 5 (3/5), showing an enlarged and thickened crest with more fat deposit in the middle and losing side-to-side flexibility, and 20% (*n* = 8) had a score of 1 out of 5 (1/5), where they showed no visual appearance of the crest but it was slightly felt through palpation. None of the horses showed a score of 0, 4, or 5 out of 5. The mean score was 1.98 and the median was 2. 

The result of the musculature atrophy evaluation was that from all horses; 52.5% (*n* = 21) showed a mild atrophy in the neck (Supplementary Figure S1) and 70% (*n* = 28) showed a mild atrophy in the hindlimbs (Supplementary Figure S2). By dividing the horses into two age groups (Group 1, <18 years; Group 2, ≥18 years), the young ones also showed a mild musculature atrophy of the neck with 42.3% (*n* = 11) and on the hindlimbs with 73.1% (*n* = 19). By comparing these two groups, in the category of severe atrophy it was more frequently measured in the elderly horses than by the younger ones.

### 3.2. Development of the New Model for Prediction of the Body Weight

The stepwise multiple regression analysis revealed that the BW of horses could be predicted with the highest accuracy and precision with the help of the data of the BCS, CNS, height at withers, circumference of the chest, cane circumference, body length, and body circumference as well as the atrophy score of the hindlimbs. The new model generated for the estimation of the BW is shown in Table 1, on the basis of which the equation for the BW prediction was established. 

**Figure 2:**
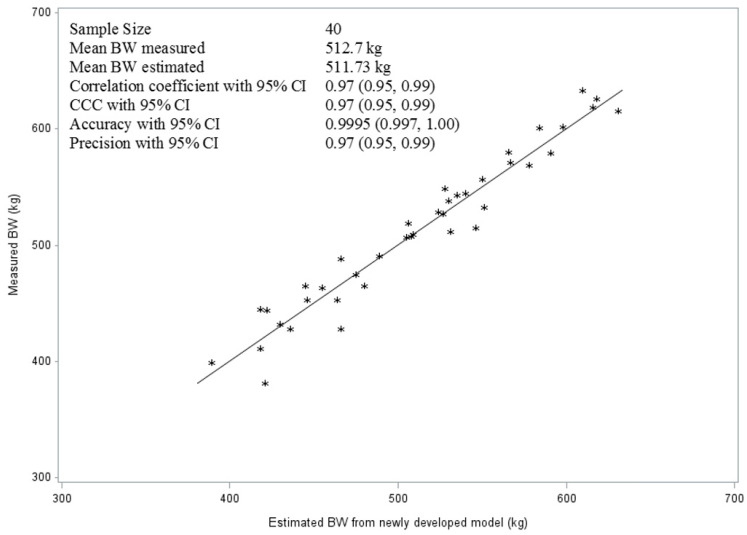
The line of best fit and the quality parameters of the model developed in this study for the prediction of body weight (BW) of horses (CCC = concordance correlation coefficient; CI = confidence intervals).

The quality of the new model was also evaluated with the help of Lin’s CCC method, using the measured BW of the horses with the scale as the gold standard. Accordingly, the mean CCC of the model as a measure of reproducibility of the gold standard with 0.97 (95% CI:0.95–0.99) can be considered as substantial (Figure 2). As indicated, the average accuracy (99.95%) was higher than the precision (97%) in the prediction of BW of the new model, though both were very high (Figure 2).

### 3.3. Evaluation of Models from the Literature

**Figure 3:**
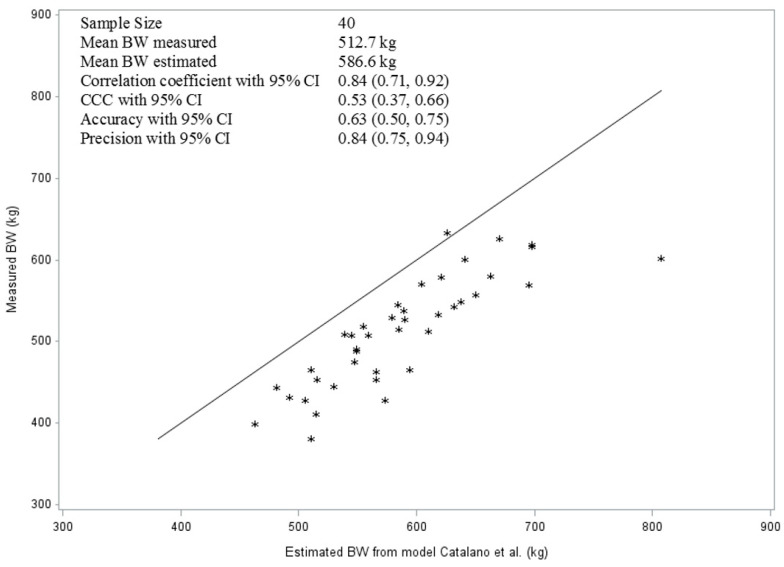
The line of best fit and the quality parameters of the model for the prediction of BW of horses using the model of Catalano et al. [20].

Again, with the help of Lin’s CCC method, we used the data of the BW to evaluate the accuracy and precision of models from the literature, such as the model of Catalano et al. [20] and that of Kienzle and Schramme [24]. Figure 3 indicates the measures of accuracy (63%) and precision (84%) of the prediction of BW of the model by Catalano et al. [20]. The mean CCC of the model as a measure of reproducibility was 0.53, only moderate. 

**Figure 4:**
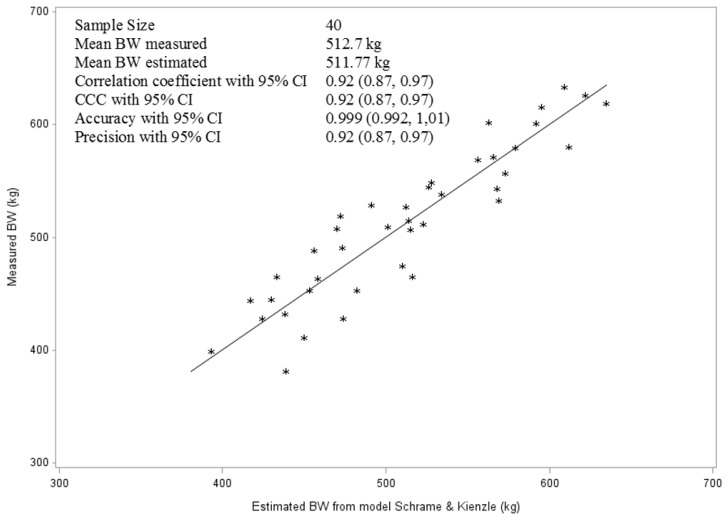
The line of best fit and the quality parameter of the model for the estimation of BW of horses using the model of Schramme and Kienzle [24].

Figure 4 indicates the measures of accuracy (99.9%), which were higher than the precision (92%) in the prediction of BW, based on the model by Schramme and Kienzle [24]. Lin’s CCC, as a measure of the reproducibility of this model, was also 0.92. By comparing the actual BW with the scale and the calculated BW using the model of Schramme [10], the result showed that in 60% (*n* = 24) of the horses the BW was underestimated, meaning the result of the calculated BW was lower than the actual one. The mean value was −18.5 kg and the span reached from −47 to −1 kg of the actual BW. In 35% (*n* = 14) of the animals, the BW was overestimated by the mean of +29.1 kg and a span from +6 to +58 kg. Only in 5% of animals were the actual and calculated values exactly the same. 

### 3.4. The Model of Prediction of the Body Condition Score and Comparison with Literature

The best model found with the help of the stepwise multiple regression analyses to predict the BCS of horses revealed that it could be predicted with the data of parameters such as age; body length; and the index consisting of measurements of the body circumference to height of withers and the atrophy of the neck. More exactly, the index is composed of the average of the chest circumference, the abdominal circumference, the neck circumference, and the flank circumference divided by 4 and its value divided by the stock size. This new model generated for the estimation of the BCS is shown in Table 2, on the basis of which the equation for the BCS prediction was established. It is evident that age, body length, and atrophy neck score have a negative effect on the BCS of the horses, whereas the index of the body circumference to height of the withers showed a positive effect. 

Comparing the BCS measured on site with the calculated BCS by the model of Laubach [25], we observed that in 42.5% (*n* = 17) of the cases the BCS was identical. In 35% (*n* = 14), the calculated BCS was underestimated, meaning the calculated results were lower than those examined on site. In this case, the value differed on average by −0.86. The BCS was overestimated in 22.5% (*n* = 9) of the horses, and the mean deviation was +0.72. 

### 3.5. Characterization of the Body Condition and Muscle Atrophy by Owners

The survey was filled out by 38 owners. The questionnaire revealed that nearly all horses were used as leisure horses (97.5%, *n* = 39), few for sporting performance (5%, *n* = 2), and only 2.5% (*n* = 1) were declared as a retirement horse. Although there was a muscle atrophy found in most of the horses, the majority of the owners (85%, *n* = 34) did not recognize it in the last six month when asked in the survey study. In the assessment of BCS, only 2.5% (*n* = 1) stated that the horse had a poor body condition, 30% (*n* = 12) indicated that this was moderate, 52.5% (*n* = 21) evaluated their horse as good, and 15% (*n* = 6) in very good body condition; in reality, for the horses found in this study, 7.5% (*n* = 3) had a poor condition, 37% (*n* = 15) were stated as moderate, and 55% (*n* = 22) had a good body condition. Furthermore, many owners stated the status of the condition stayed constant (62.5%, *n* = 25) and only 37.5% (*n* = 15) stated that it was variable during the last six months. 

## 4. Discussion

One hypothesis of this study is that the average BCS of the horses is not in the optimal range of 5 according to the scoring system of Henneke [8]. Using the system of Henneke [8], the results showed that 55% (*n* = 22) of the horses did not show an optimal BCS of 5/9 but fell into the categories above (BCS > 5/9). Furthermore, our study showed that younger horses are more affected by over-conditioning (69.2%, *n* = 18). This indicates an oversupply of energy relative to the animal’s requirements, which seems to be particularly relevant in younger horses. This finding agrees with previous observations in the literature [1,2,3] and emphasizes the need for monitoring of BCS of horses in practice to avoid over-conditioning and the diseases related to it. Although this result was expected, one must keep in mind here when interpreting the results that the classification system used by Henneke [8] has discrepancies. Firstly, it was developed for Quarter Horse mares and is not ideally suited for Warmblood horses [10]; in addition, it is not entirely optimal for obese horses [29,30]. In this study, not only were different breeds of horses represented, including Coldblood horses and breed mixes, but some animals also fell between two scores, which made classification using this system difficult. Therefore, half points were assigned, which statistically rounded up these horses and automatically assigned them to the higher score. For further research, a classification system for Coldblood horses and other breeds would be interesting, as well as a system that allows half points/scores to be awarded. 

Another interesting finding of this study is that horse owners seem to have little awareness of the expression of their horse’s musculature, as 85% of the participants did not notice any muscle atrophy. However, the results of this study show that 52.5% (*n* = 21) of the horses had mild muscle atrophy in the neck and 70% (*n* = 28) of horses had mild muscle atrophy in the hindlimbs. Basically, there are a number of causes that can lead to muscle atrophy, for example, the feed composition [16]; the factor of aging, where a shift of muscle fiber type composition and a decrease in mitochondrial density appears [14]; as well as chronic disease like Polysaccharide Storage Myopathy Type 1 (PSSM1 homozygous), Pars Pituitary Intermedia Dysfunction (PPID), as well as age-related inflammation (Inflamm-aging) [15,31]. In this study, the causes of muscle atrophy were not explored; so, to fully elucidate the muscle atrophy in these horses, a re-analysis of the feed ration, the medical history of each animal, and the analysis of training and possibly other aspects is needed. Moreover, currently, there is no comparative literature where the muscle atrophy score of horses in practice has been determined to compare with the data collected. Therefore, further research is needed, especially in the field of sport horses, to determine if these animals show atrophy and how it relates to their exercise, performance, and feeding. 

By taking a look at the BW models, it stands out that only the body length is represented in the equation. Hereby, it becomes clear that not only does the body length have a poor repeatability but it is also dependent on the horse’s hindlimb position. Furthermore, it is very difficult to determine the beginning and end point by measuring the body length if the horse has a good expression of musculature [10]. That could be the reason why existing BW models did not fit accurately; because of that, not only the body length but also the body circumference are included in the newly developed model of this study. Besides, body condition and fat reserves play an important role in estimating the BW. The model by Catalano et al. [20] does not take them into consideration, whereas the model by Kienzle and Schramme [24] accounts only for the BCS of the horses. Both variables, the BCS and the CNS, were included in the new model, which likely improved the quality of the prediction of the model. Since the height at the withers and the cane circumference have good repeatability [10], these two parameters were kept as variables in other models. To achieve an even more accurate measurement, a tape measure could be used for measurement instead of the stock measurement used in this study, which is also better tolerated by the horses and owners [10]. Another improvement in our model is that the abdominal circumference and the atrophy of the hindlimb were also taken into account to estimate the BW. This might have improved the quality indicators of the model further, such as reproducibility and accuracy, because the expression of musculature is of great relevance for the BW [18]. Therefore, it also should be recognized that 70% of the participating horses in the study represented an atrophy of the hindlimb. So, for that reason, the new model should be checked for accuracy on the basis of a greater animal number, which have a good, pronounced musculature. With regard to the abdominal circumference, it should be noted that it also may reflect the intestinal filling status; however, weight loss can still be detected with it [32]. As many as 65% (*n* = 26) of the horses in this study had 24-h access to hay, allowing a constant feed intake. The passage through the stomach and small intestine is approximately five hours, which, in contrast, can be up to 35 h through the large intestine [33]; so, multiple measurements throughout the day, rather than immediately after feeding, would be useful to minimize error. In restrictively fed horses, the feeding time may be taken into consideration before measuring the abdominal circumference. Since the sample size of 40 horses is considered to be limited, the new model should be validated using a larger number of animals, including various breeds, ages, and muscularity.

By taking a look at the hay quality, the result showed that 38.5% of the samples were in good and 38.5% in a satisfactory nutritive value (hay quality), and a moderate one represented only 23.08% (*n* = 3) of the samples. In contrast to the result, by asking the owners to estimate the quality, they tendentially overestimated it, reporting the hay of 29% as very good, of 39.5% as good, and of 29% as satisfactory. In contrast, in another study, which took place in Luxemburg, the participants underestimated the hay quality compared to the quality checks [25]. In order to find out why the horse owner under- or overestimates the quality, more questions should be asked as to what criteria the opinion is based on to determine the classification. On Austrian farms, hay with many stalks and few leaves is often found, which thus has unfavorable digestibility and reduced metabolic energy concentration; further, up to 30% forage contamination by soil is found [34], which excludes very good hay quality. The differences in hay quality could be explained by the fact that in this study the samples were only taken at one point in time, thus reflecting a snapshot, and this was also limited to the Lower Austria area only. In order to obtain a more representative result of the hay quality, the samples would have to be taken several times and with longer time intervals. 

This research also has some limitations, which need to be taken into consideration in future studies. The first limitation is that the prediction equations were developed based on a limited sample size. Therefore, it is necessary to validate these equations with larger and more diverse cohorts of animals in future studies. Additionally, although the muscle atrophy scoring system was shown to be a strong indicator of the degree of muscularity and hence of the BW and BCS of the animal, such scoring systems can be subjective and influenced by observer bias, potentially leading to variations in the results obtained by different observers. Therefore, future research should explore more objective and quantitative methods, such as imaging, histological analysis, or molecular markers, to assess muscle atrophy and provide more reliable outcomes. While we have taken into account various morphometric measurements and scoring parameters as predictors, there might still be other important factors not considered in the equations (i.e., breed, the degree of muscularity, exercise, feeding time, and gut fill) that could impact BW and BCS prediction. Future investigations could explore additional potential predictors to further enhance the accuracy of the prediction models. Moreover, although the research already considered the gender of the horses (mares and geldings) as a predictive factor, there may be gender-related differences specific to different horse breeds that warrant further exploration in future studies.

## 5. Conclusions

In conclusion, the study revealed both over-conditioning (55% of the horses in this study showed a BCS of 6 to 7 on a scale to 9) and muscle atrophy at the neck (52.5%) and at the backhand (70%) in leisure horses; yet, in all cases, the horse owners were only minimally aware of these. By comparing the animals’ actual body weight and the body weight calculated by models in the literature, it was found that 60% of the horses have on average a higher body weight (+18.5 kg) than the calculated value and the determined BCS agrees with the calculated BCS only in 42.5% of the cases. Most importantly, this research developed new models to estimate both the body weight and the BCS of horses using common morphometric measurements of the body size, such as the neck as an indication of regional adiposity and the muscle atrophy score as a guide for the degree of the muscularity of the horse, as well as the age of the animal. Eventually, taking into account all these parameters increased the accuracy, precision, and reproducibility of the models as compared with previous ones from the literature. Future research should use larger cohorts of animals—in particular, those with a high variety regarding the degree of muscularity and physical activity—to validate the findings of this study. 

## Figures and Tables

**Table 1:** The best-fit estimated parameters of the model for the estimation of body weight (BW) of horses based on various morphometric variables (Root MSE = 17.86, R^2^ = 0.94, Adjusted R^2^ = 0.93, and Variation Coefficient = 3.48).

Variable	Estimated Parameter	SE	*p*-Value	VIF
Intercept	−1028.9938	86.73	<0.001	0
Body Condition Score	19.96658	7.09	0.0084	2.48
Cresty Neck Score	11.87471	6.68	0.0853	1.78
Height of the withers (cm)	1.68772	0.62	0.0103	2.20
Abdomen circumference (cm)	1.13782	0.48	0.0237	3.34
Cane circumference (cm)	3.5439	1.71	0.0468	1.76
Body length (cm)	0.56605	0.19	0.0063	1.43
Body circumference (cm)	1.90782	0.38	<0.001	4.13
Atrophy score of the hindlimb	−17.46599	6.18	0.0081	1.37

SE = standard error; VIF = variance inflation factor.

**Table 2:** The best-fit estimated parameters of the model for the estimation of body condition score (BCS) based on several body metrics and the age of horse (Root MSE = 0.41, R^2^ = 0.58, Adjusted R^2^ = 0.58, and Variation Coefficient = 7.55).

Variable	Estimated Parameter	SE	*p* Value	Variance Inflation
Intercept	1.81445	1.57	0.2543	0
Age (years)	−0.04016	0.01	0.0038	1.13
Index ^1^	5.26108	1.22	0.0001	1.10
Body length (cm)	−0.00678	0.004	0.0891	1.07
Atrophy Neck Score	−0.34174	0.12	0.0061	1.15

SE = standard error. ^1^ $Index=\frac{\left(\frac{Chest\;circumference\;\left(cm\right)\;+\;Abdomen\;circumference\;\left(cm\right)\;+\;Neck\;circumference\;\left(cm\right)\;+\;Flank\;circumference\;(cm)}{4}\right)}{Height\;of\;the\;withers\;(cm)}$.

## Data Availability

Not applicable.

## Supplementary Materials

The following supporting information can be downloaded at https://www.mdpi.com/article/10.3390/vetsci10080515/s1, Figure S1: Muscle Atrophy Scores of the Neck by two Age Groups; Figure S2: Muscle Atrophy Scores of the Hind Limbs by two Age Groups; Table S1: Descriptive statistics of data of the body weight (BW) and of the morphometric measurements of the horses.
