# Supplementary material for: Morphometric Measurements and Muscle Atrophy Scoring as a Tool to Predict Body Weight and Condition of Horses
Source: Vet Sci. 2023 Aug 9;10(8):515. doi: 10.3390/vetsci10080515 (PMC10458044; doi:10.3390/vetsci10080515)
Supplement: Supplementary file 1 [file vetsci-10-00515-s001.zip › vetsci-2499911-Supplementary Table S1-done.pdf]

veterinary sciences

MDPI

Supplementary Table S1 Descriptive statistics of data of the body weight (BW) and of the morphometric measurements of the horses

Variable	n	Span	Mean	Median	SD
BW all horses	40	381–633	512.7	513.3	66.96
BW horses < 18 years	26	428–633	528	533.6	62.2
BW horses ≥ 18 years	14	381–557	473.8	464.0	59.08
Hight at the withers	40	150–175	158	157	6.87
Neck length	40	59–81.5	67.65	67.75	4.62
Neck circumference	40	102–163	123.9	123.0	9.48
Crest of the neck	40	2.5–12.0	4.58	4.00	1.99
Chest circumference	40	172.5–209.0	188.6	189.2	9.06
Abdomen circumference	40	187.5–234.0	209.4	209.2	10.93
Flank circumference	40	175.0–233.0	198.6	199.0	12.94
Cane circumference	40	18.5–27.0	21.35	20.75	2.22
Body length	40	152–274	173.6	171.5	17.68
Body circumference	40	375–433	399.5	398.0	15.27

n = number of horses

SD = standard deviation

BW in kg

All measurements in cm
