# Supplementary figures and images for: Morphometric Measurements and Muscle Atrophy Scoring as a Tool to Predict Body Weight and Condition of Horses
Source: Vet Sci. 2023 Aug 9;10(8):515. doi: 10.3390/vetsci10080515 (PMC10458044; doi:10.3390/vetsci10080515)

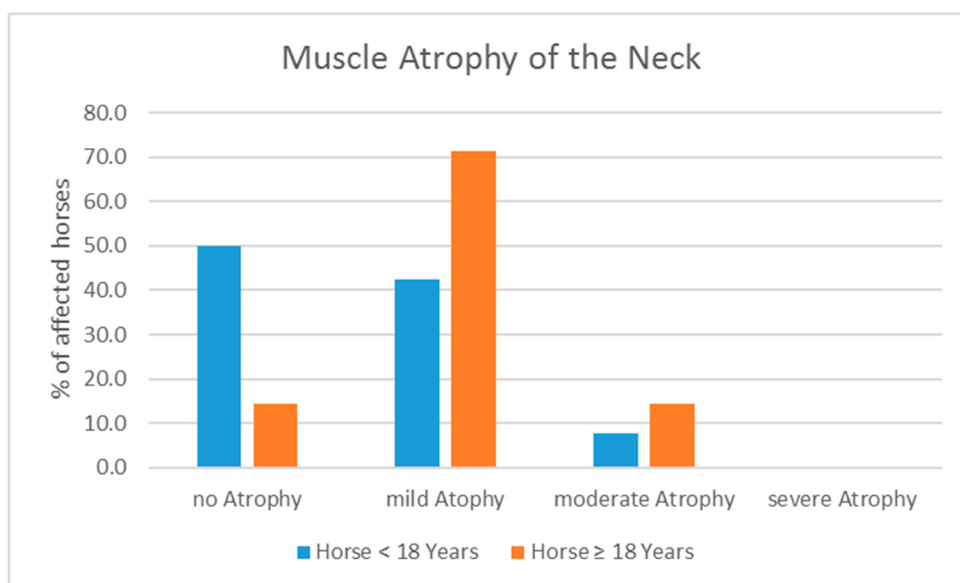

Supplementary Figure S1: Muscle Atrophy Scores of the neck by two age groups

Supplement: Supplementary file 1 [file vetsci-10-00515-s001.zip › vetsci-2499911-Supplementary Figure S1-done.pdf]

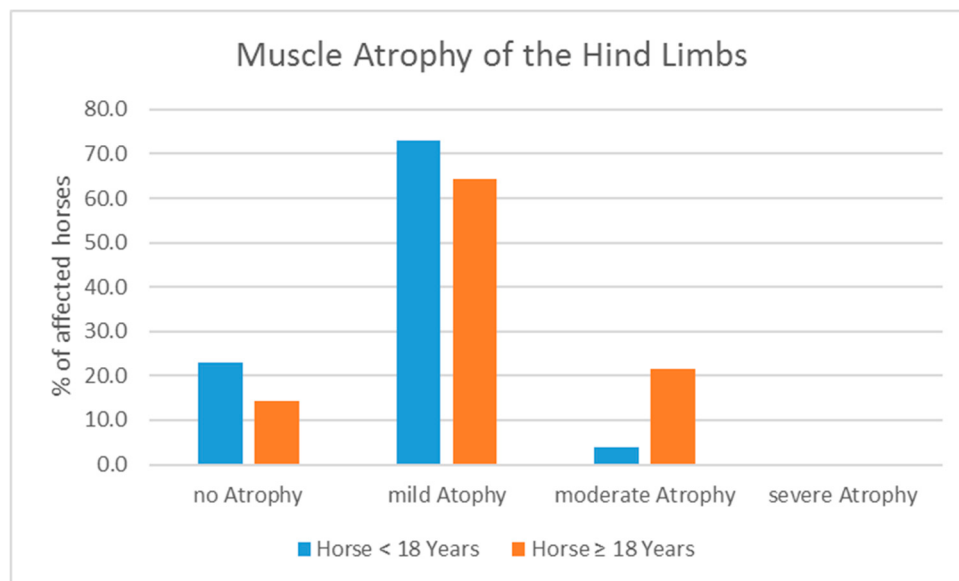

Supplementary Figure S2: Muscle Atrophy Scores of the hind limbs by two age groups

Supplement: Supplementary file 1 [file vetsci-10-00515-s001.zip › vetsci-2499911-Supplementary Figure S2-done.pdf]
